# Classification of neurological abnormalities in children with congenital melanocytic naevus syndrome identifies magnetic resonance imaging as the best predictor of clinical outcome

**DOI:** 10.1111/bjd.13898

**Published:** 2015-08-27

**Authors:** R. Waelchli, S.E. Aylett, D. Atherton, D.J. Thompson, W.K. Chong, V.A. Kinsler

**Affiliations:** ^1^ Paediatric DermatologyGreat Ormond St Hospital for ChildrenLondonU.K.; ^2^ NeurosciencesGreat Ormond St Hospital for ChildrenLondonU.K.; ^3^ Neurosciences UnitUCL Institute of Child HealthLondonU.K.; ^4^ Paediatric NeurosurgeryGreat Ormond St Hospital for ChildrenLondonU.K.; ^5^ Paediatric NeuroradiologyGreat Ormond St Hospital for ChildrenLondonU.K.; ^6^ Genetics and Genomic MedicineUCL Institute of Child HealthLondonU.K.

## Abstract

**Background:**

The spectrum of central nervous system (CNS) abnormalities described in association with congenital melanocytic naevi (CMN) includes congenital, acquired, melanotic and nonmelanotic pathology. Historically, symptomatic CNS abnormalities were considered to carry a poor prognosis, although studies from large centres have suggested a much wider variation in outcome.

**Objectives:**

To establish whether routine MRI of the CNS is a clinically relevant investigation in children with multiple CMN (more than one at birth), and to subclassify radiological abnormalities.

**Methods:**

Of 376 patients seen between 1991 and 2013, 289 fulfilled our criterion for a single screening CNS MRI, which since 2008 has been more than one CMN at birth, independent of size and site of the largest naevus. Cutaneous phenotyping and radiological variables were combined in a multiple regression model of long‐term outcome measures (abnormal neurodevelopment, seizures, requirement for neurosurgery).

**Results:**

Twenty‐one per cent of children with multiple CMN had an abnormal MRI. Abnormal MRI was the most significant predictor of all outcome measures. Abnormalities were subclassified into group 1 ‘intraparenchymal melanosis alone’ (*n* = 28) and group 2 ‘all other pathology’ (*n* = 18). Group 1 was not associated with malignancy or death during the study period, even when symptomatic with seizures or developmental delay, whereas group 2 showed a much more complex picture, requiring individual assessment.

**Conclusions:**

For screening for congenital neurological lesions a single MRI in multiple CMN is a clinically relevant strategy. Any child with a stepwise change in neurological/developmental symptoms or signs should have an MRI with contrast of the brain and spine to look for new CNS melanoma.

## Abstract 2

**What's already known about this topic?**

- Multiple congenital melanocytic naevi (CMN; more than one lesion at birth) can be associated with abnormalities of the central nervous system (CNS). The spectrum of these abnormalities includes congenital and acquired pathologies, melanotic and nonmelanotic lesions, rendering the term ‘CMN syndrome’ more appropriate than ‘neurocutaneous melanosis’.
- Symptomatic CNS abnormalities were previously thought to carry a universally poor prognosis, although cohort data in the last decade have argued against this.

**What does this study add?**

- A single CNS magnetic resonance imaging scan in multiple CMN, independent of projected adult size or site of the largest naevus, and ideally in the first 6 months of life, is currently an appropriate screening strategy.
- An abnormal result is a better statistical predictor of clinical outcome than cutaneous phenotype.
- Clinical management is altered as a result of the radiological result.

Congenital melanocytic naevi (CMN) can be single or multiple at birth. Multiple CMN, defined as more than one CMN at birth, can be associated with neurological abnormalities of the central nervous system (CNS), traditionally termed neurocutaneous melanosis (NCM). Patients with multiple CMN also have an increased risk of primary melanoma developing in the CNS or in the skin.^1^ The underlying cause of multiple CMN and so‐called NCM was recently found to be mosaicism for heterozygous activating mutations in codon 61 of *NRAS,* a developmental gene and oncogene involved in the control of key cell signalling pathways.^2^ However, the onset of melanoma requires further genetic events.^2^

Although neurological abnormalities are well established as the most common extracutaneous manifestations of mosaicism in children with multiple CMN, with an incidence ranging from 10% to 33% in clinical studies,^3^, ^4^, ^5^ the blanket term ‘NCM’ has been applied to all abnormalities, with no systematic subclassification. This term was originally proposed by Rokitansky in 1861 as a description of autopsy findings in a single case with fatal melanotic leptomeningeal disease, which we would now recognize as melanoma. With the advent of magnetic resonance imaging (MRI) and the description of the characteristic signal for melanin,^5^, ^6^ the spectrum of described neurological abnormalities has expanded to include congenital and acquired, melanotic and nonmelanotic lesions, with widely varying clinical outcomes ranging from benign quiescent lesions to fatal malignancy. The most common abnormality on MRI in either asymptomatic populations or prospectively collected populations is isolated intraparenchymal melanosis (foci of melanin‐containing cells in the brain parenchyma),^3^, ^7^ previously thought to be secondary only to overlying invasive leptomeningeal disease. Although this can occur in the context of malignant disease, several histopathological studies have proven the presence of congenital melanotic parenchymal deposits without involvement of the overlying meninges. The melanin in these lesions is produced within neurons and glia rather than melanocytes, and there are subtle signs of focal cortical dysplasia within these lesions.^8^, ^9^, ^10^, ^11^, ^12^, ^13^

Other less frequent neurological diagnoses include syringomyelia, nonmalignancy‐related hydrocephalus, tumours (including ependymoma, meningioma, astrocytoma, choroid plexus papilloma and pineal germinoma) and malformations such as Dandy–Walker and Arnold–Chiari malformations.^5^, ^6^, ^13^, ^14^, ^15^ The risk of congenital neurological abnormalities in children with CMN increases with the size of the largest CMN and the total number of naevi.^3^, ^4^, ^16^ These two variables are intimately but complexly connected, with the increasing size of the largest naevus usually but not always associated with increasing numbers of total naevi. As such, these variables confound each other within logistic regression models where both are used. As both these measures are relatively inaccurate it is difficult to say with confidence which is most reliable, but, in our experience, the projected adult size of the largest lesion is a more robust measurement in statistical models.

There have been other confounders in the study of neurological abnormalities in CMN. For example, CMN distribution over the posterior axis (overlying the head, neck or spine) is no longer considered to be a risk factor for neurological abnormalities but rather a confounder for size of the main CMN.^3^, ^17^, ^18^ Furthermore, primary CNS melanoma can develop either in the parenchyma or in the leptomeninges, and this data has usually been amalgamated with data on congenital abnormalities. Where primary melanoma occurs the clinical picture is of sudden clinical deterioration, usually with symptoms of raised intracranial pressure and/or of spinal compression.

It is commonly suggested in the literature that the outcome of children with ‘symptomatic NCM’ is extremely bleak,^16^, ^19^ with near‐certain mortality. However, more recent larger studies have reported symptoms in many individuals with MRI abnormalities where the outcome has not been fatal.^3^, ^14^, ^15^ The original perception is based partly on reports of neurological involvement prior to the advent of MRI, all of which were at autopsy and were therefore likely malignant processes rather than congenital abnormalities, and partly on a lack of large prospective studies of children with this rare condition.

The primary aim of this study was therefore to subclassify the CNS congenital radiological abnormalities on the first screening scan in a large cohort of children with CMN, and to correlate these findings with clinical outcome measures. A secondary aim was to re‐evaluate our 2008 guidelines for imaging of the CNS in order to assess whether these have proved clinically useful over the last 6 years.

## Patients and methods

### Patients

**Figure 1:**
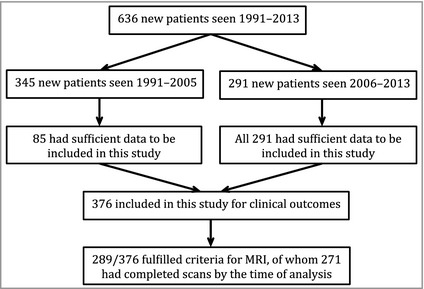
Flow chart of numbers and timings of patients seen and included in this study. MRI, magnetic resonance imaging.

Patients in this cohort were seen sequentially in the paediatric dermatology department at Great Ormond Street Hospital between 1991 and 2013, and followed prospectively. Therefore, this study includes the majority of patients studied in two previous publications by our group,^3^, ^20^ and, owing to the larger patient cohort and systematic sequential collection of patients in recent years, supplants the previous studies from the point of view of recommendations. All patients with an abnormal MRI scan were also evaluated by a paediatric neurologist and followed prospectively, which included a detailed neurodevelopmental and neurological history and examination. In total, 636 new patients were seen (see Fig. 1), of whom 376 were deemed after a review of the notes to have had sufficient clinical data collected at the first visit for them to be included in this analysis. The total of 376 includes all new patients (*n *=* *291) seen sequentially since 2006, after a standardized clinical data collection form was introduced. In total, 289 of the 376 patients fulfilled our criteria for MRI of the CNS, and 271 had successfully completed scans at the time of analysis. The MRI scans were undertaken almost exclusively under sedation only, rather than under general anaesthesia, as is standard practice in our hospital for children < 1 year of age. Where scans were not completed this was due to either failure of sedation of the patient or failure to attend for the appointment on two occasions. Images were analysed by a paediatric neuroradiologist with expertise in CMN, who was aware of the diagnosis of multiple CMN but who did not have details on clinical outcomes.

### Inclusion criteria

Before 2008 the criteria for MRI were a CMN > 2 cm overlying the spine or brain, or a CMN elsewhere of at least the size of the patient's hand. After results published in 2008,^21^ the criteria were changed to include only those with multiple CMN (i.e. more than one at birth) independent of the site and size of the lesions, if presenting before the age of 2 years. If the patient is older than 2 years at first presentation and neurologically normal we do not perform a screening MRI as most congenital lesions requiring treatment should have declared themselves by this stage. Urgent MRI is performed at any age with any CMN if neurological symptoms occur. Only four children were neurologically symptomatic at the time of the referral to our department.

### Outcomes

The phenotypic variables analysed were radiological (MRI result and subsequent classification) and clinical (largest CMN projected adult size, total number of naevi at birth and at enrolment). Adverse outcome measures were seizures, neurodevelopmental problems, whether neurosurgery was required, primary CNS malignant melanoma, and death from primary CNS melanoma or primary cutaneous melanoma. Neurodevelopmental problems were defined as those diagnosed by a paediatrician or a paediatric neurologist, or officially by school assessment and requiring an educational intervention.

Nonfatal melanoma was not included as an outcome measure as there were no cases in this cohort where melanoma was not fatal. Statistical modelling of outcome measures was performed by multiple logistic regression, considering the whole cohort together. For statistical analysis of the effect of guidelines introduced in 2008, the frequency of scans ordered and the percentage of abnormal scans was compared in the pre‐ and post‐2008 cohorts by a two‐tailed Fisher's exact test.

## Results

In total, 289 children fulfilled the criteria for MRI of the whole CNS. Two hundred and seventy‐one of these scans were performed successfully. The mean and median ages at time of first MRI were 1·5 and 0·6 years, respectively (SEM 0·2), and the mean and median length of follow‐up was 11·0 and 8·5 years, respectively (SEM 0·4). The cutaneous phenotype spectrum of this tertiary referral centre cohort is skewed towards the severe end, with 62% of the patients having a CMN of > 20 cm projected adult size, and 41% of > 40 cm (Table 1).

**Table 1:** Frequencies of different cutaneous phenotypic features in the study cohort

Projected adult size of largest naevus (cm)	*n *=* *265
< 10	42 (15·9)
10–20	50 (18·9)
20–40	55 (20·8)
40–60	46 (17·4)
> 60	64 (24·2)
No one larger lesion^a^	8 (30·2)
Site of largest CMN	*n *=* *157
Face	9 (5·7)
Scalp	19 (12·1)
Trunk	82 (52·2)
Limb	18 (11·5)
Scalp/neck/trunk	13 (8·3)
Face/scalp	12 (7·6)
No one larger lesion^a^	4 (2·6)
Total number of other naevi at birth (previously termed satellites)^b^	*n *=* *165
0	14 (8·5)
< 10	54 (32·7)
10–20	33 (20·0)
20–25	30 (18·2)
50–100	18 (10·9)
100–200	10 (6·1)
> 200	6 (3·6)

Data are *n* (%). CMN, congenital melanocytic naevi. ^a^Individuals with ‘no one larger lesion’ do not have one naevus clearly bigger than all the others but rather a collection of similar‐sized lesions. This phenotype used to be called ‘multiple CMN’ but owing to the normal understanding of the word ‘multiple’ we think this term is confusing and best avoided in this context. ^b^The term ‘satellite naevus’ is sometimes used to mean any other naevus on a patient with CMN which was not the largest naevus. We no longer use this term as it implies some sort of hierarchy and geographical relationship between the largest naevus and the smaller naevus,^22^ and all these are, in fact, CMN that have arisen from the same postzygotic mutation and may not necessarily be close to each other. Within the frustrating limits of the current, relatively inaccurate classification system for CMN we therefore prefer to count the total number of naevi.

Overall, 46 of 271 (17%) MRIs from the whole cohort were abnormal, rising to 21% in the post‐2008 criteria cohort (nonsignificant difference; see below for comparison of pre‐ and post‐2008 data). Abnormalities were subclassified into group 1, ‘intraparenchymal melanosis alone’ (*n *=* *28), as this is the most common single finding in patients with CMN, and group 2, ‘other CNS pathology’ (*n *=* *18). Group 2 was too small to subdivide for analysis, as it included a wide variety of different CNS pathologies (Table 2).

**Table 2:** Clinical and radiological features of the 18 patients with abnormal magnetic resonance imaging (MRI) scans (classified as group 2, ‘other pathology’)

Patient	Sex	CMN projected adult size (cm)	Total naevi at enrolment (*n*)	MRI findings at first scan, with subsequent progress	Neurological symptoms by time of first MRI	Seizures ever	Neurodevelopmental problems (ever)	Neurodevelopmental details	Neurosurgery	Death from cutaneous melanoma	Death from CNS melanoma
1	M	> 60	20–50	Generalized lack of white matter bulk, slightly prominent ventricles, low‐volume inferior vermis Appearances stable on repeat MRI (×1), neurologically normal at age 8 years	No	No	No	–	No		
2	M	> 60	100–200	Multiple foci of intraparenchymal melanosis amygdala, temporal lobe and cerebellum Right cerebellar hemisphere small Appearances stable on repeat MRI (×1) ADHD diagnosed at age 4 years	No	No	Yes	ADHD, mild developmental delay, speech therapy, extra help in class, mainstream school	No		
3	M	20–40	20–50	Leptomeningeal enhancement over anterior surface of conus and along the nerve roots of the cauda equina Appearances stable on repeat MRI (×2), neurologically normal at age 4 years	No	No	No		Yes		
4	F	10–20	< 10	Filum terminale lipoma Lateral ventricles large right > left but not hydrocephalus; some lack of white matter bulk and cortical thinning Appearances stable on repeat MRI (× 1), neurologically normal at age 10 years	No	No	No		No		
5	F	40–60	> 200	Multiple foci of intraparenchymal melanosis amygdala, cerebellum; two benign intradural tumours (one melanocytic, one neurocristic hamartoma), DWM hydrocephalus, later syrinx within spine Multiple MRI scans, DWM resolved, diffuse leptomeningeal disease (melanocytic on histology but no characteristic melanin signal on MRI) spread over first year to involve all leptomeninges, cord compression gradually worsening In wheelchair with moderate global developmental delay at age 10 years	No	Yes	Yes	Moderate global developmental delay, wheelchair bound	Yes		
6	M	10–20	2	Right lateral ventricle choroid plexus papilloma, hydrocephalus	No	Yes	No		Yes		
7	F	Multiple	10–20	Multiple foci of intraparenchymal melanosis plus multiple focal nonmelanin signal spinal thoracic dural deposits Not biopsied, stable on repeat MRI (×2), neurologically normal at age 5 years	No	No	No		No		
8	F	> 60	50–100	Extramedullary dural stranding	No	No	No		No		
9	M	< 10	Multiple but exact number unknown	Right cerebellar astrocytoma	No	No	No		Yes		
10	M	20–40	> 200	Intraparenchymal melanosis of both thalami and mesial temporal lobes (left > right), venous angioma left cerebellar hemisphere	No	Yes	Yes	Moderate cognitive delay, special school	Yes		
11	M	40–60	100–200	Intraparenchymal melanosis of left amygdala Equivocal enhancement of nerve roots of cauda equina	No	No	No		No		
12	Male	40–60	Multiple but exact number unknown	Midline posterior fossa arachnoid cyst, communicating hydrocephalus	No	Unknown	Unknown		Yes		
13	M	10–20	< 10	Left frontal lobe meningioma	No	No	No		Yes		
14	M	10–20	< 10	Subtle enhancement of nerve roots of cauda equina	No	No	No		No		
15	M	> 60	20–50	Multiple foci of intraparenchymal melanosis and diffuse leptomeningeal melanoma	No	No	Yes		Yes	Yes	
16	M	–	Multiple but exact number unknown	Posterior fossa malignant melanoma	Yes	Unknown	Yes		Yes		Yes
17	F	No single larger lesion Largest lesion projected adult size < 5 cm	> 200	Diffuse leptomeningeal melanosis and DWM with hydrocephalus at 2 weeks At 6 months diffuse leptomeningeal melanoma with focus of intraparenchymal melanoma	No	Yes	Yes	Moderate global delay	Yes		Yes
18	F	> 60	20–50	Intraparenchymal melanosis and subsequent diffuse leptomeningeal melanoma with hydrocephalus	Yes	No	Yes	Mild developmental delay prehydrocephalus	Yes		Yes

CMN, congential melanocytic naevi; CNS, central nervous system; M, male; ADHD, attention deficit hyperactivity disorder; F, female; DWM, Dandy–Walker malformation.

Any radiological abnormality (as a binary variable, normal vs. abnormal MRI) was a stronger predictor than projected adult size for all outcome measures: seizures [odds ratio (OR) 13·4, 95% confidence interval (CI) 4·7–38·2], neurodevelopmental problems (OR 3·0, 95% CI 1·3–7·0) and requirement for neurosurgery (OR 71·0, 95% CI 8·9–567·3) (Table 3). CNS melanoma and death were not modelled as the numbers are low. When projected adult size of the largest CMN was included in these models as a categorical variable (< 10, 10–20, 20–40, 40–60 and > 60 cm), the variable as a whole was only significant for neurodevelopmental abnormalities (*P *=* *0·03). Sex (as a binary variable, male = 1) was not found to be significant in these models. For the full regression analysis, please see Appendix S1 (Supporting Information).

**Table 3:** Frequencies and odds ratios for each of the adverse clinical outcomes with respect to radiological findings. Where the numbers do not equal the total numbers for the radiologically classified group this is due to missing data for a very few patients on outcomes

	Normal MRI	Intraparenchymal melanosis only	Other pathology	OR (95% CI) for modelling these outcomes by MRI result (binary − normal/abnormal)
Neurodevelopmental problems	26/226 (11·5%)	8/28 (28·6%)	7/17 (41·2%)	3·0 (1·3–7·0)
Seizures	6/220 (2·7%)	7/27 (25·9%)	4/16 (25%)	13·4 (4·7–38·2)
Requirement for neurosurgery	1/226 (0·4%)	0/27 (0%)	11/8 (61·1%)	71·0 (8·9–567·3)

Data are *n* (%) unless otherwise indicated. MRI, magnetic resonance imaging; OR, odds ratio; CI, confidence interval.

**Figure 2:**
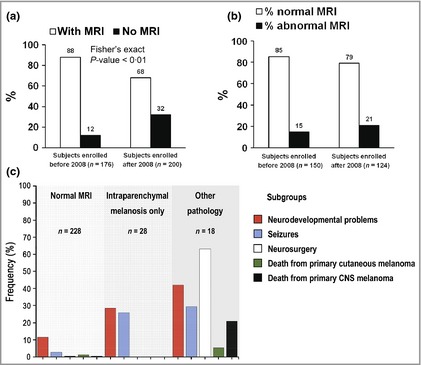
(a) Comparison of the percentage of patients in whom a magnetic resonance imaging (MRI) scan was performed (white column) and not performed (black column) before and after 2008. By excluding those with only a single congenital melanocytic naevus (CMN), independent of size or site, the introduction of guidelines in 2008 has significantly reduced the percentage of patients scanned routinely. However, the percentage of abnormal scans is not significantly altered, suggesting that we have become more efficient at detecting the same rate of abnormalities. (b) Comparison of the percentage of patients with a normal MRI result (white column) with those with an abnormal result (black column) before and after 2008. The introduction of guidelines in 2008 has not significantly altered the percentage of abnormal scans detected, which implies that we are not failing to detect significant numbers of abnormalities. (c) Subclassification of the radiological abnormalities in this cohort of children with CMN and correlation with the incidence of the different clinical outcome measures in each group. CNS, central nervous system.

Seizures and abnormal neurodevelopment were seen in a minority of the normal MRI group; however, not only were these numbers small, but the seizures were also a temporary problem and/or easy to control with a single medication, and the neurodevelopmental abnormalities were mild, compatible with normal schooling. This is a notably milder clinical phenotype than that seen in those with seizures or neurodevelopmental abnormalities in the other radiological groups (Table 3). Of note, one patient with a normal initial MRI scan developed primary CNS melanoma requiring neurosurgery and later died from primary CNS disease (Fig. 2). This patient was first scanned in the late 1990s and the quality of MRI has improved since then. Therefore, it is possible that there was a neurological disease present that was below the resolution of scanning, as has been shown histologically.^13^

Of those with abnormal scans, there was a striking difference between the two subgroups in clinical outcomes. While a substantial proportion of patients in group 1 had neurological symptoms, namely 25·9% with seizures, and 28·6% with neurodevelopmental abnormalities (not significantly different from group 2), there were no patients with melanoma in this subgroup, and no deaths. Although CNS melanoma must be possible in this group the risk appears to be low in childhood. Furthermore, we can conclude that in patients with intraparenchymal melanosis alone, symptoms can be related to that congenital disease and do not necessarily equate with malignancy or death. Therefore, with classical radiological features intraparenchymal melanosis alone does not require surgical intervention. Because of this subtlety – the differentiation between symptomatic congenital disease and new onset of symptoms from CNS melanoma – we would continue to recommend a repeat MRI of the CNS in any child who presents with new neurological symptoms. This can then be compared with baseline scans to look for new lesions or progression.

**Figure 3:**
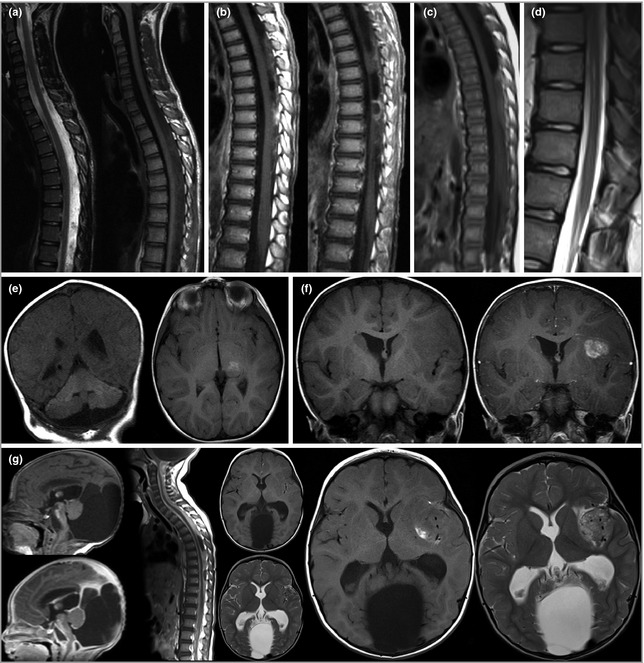
(a) Intradural extramedullary disease, presumed leptomeningeal, dorsal to the spinal cord. (b) Enhancing intradural extramedullary disease, presumed leptomeningeal, dorsal to the spinal cord. (c) Intradural extramedullary disease, presumed leptomeningeal, dorsal to the spinal cord. (d) Prominent central canal of the lower thoracic cord. These lesions have not been biopsied and therefore no exact diagnosis is available. However, in all cases shown the patients do not exhibit spinal symptoms or signs, and magnetic resonance imaging (MRI) appearances have been stable since birth, with follow‐up now at ages 5, 6, 10 and 16 years. (e) Extensive intraparenchymal melanosis of the cerebellum and focal melanosis of left thalamus. (f) Pre‐ and postgadolinium‐enhanced MRI showing meningioma in left sylvian fissure. (g) Pre‐ and postgadolinium‐enhanced MRI showing Dandy–Walker malformation with congenital leptomeningeal disease at 13 days. Further progression of leptomeningeal disease at 22 months. Intraparenchymal melanoma of left sylvian fissure at the age of 24 months, which appears to have developed from leptomeningeal infiltration, although this is not always the case.

The clinical outcome pattern in group 1 was different to that of group 2, where both the requirement for neurosurgery and the mortality rate from CNS melanoma was substantial (Table 3). The requirement for neurosurgery in group 2 was significantly higher compared with group 1 (Fisher's exact test *P *<* *0·01), but the mortality rate from CNS melanoma did not differ, perhaps owing to low numbers (Fisher's exact *P *=* *0·06). However, group 2 was very heterogeneous. The clinical symptoms in this subgroup ranged from none to mild speech delay to severe global delay (Table 2). Three of the 18 patients presented with an isolated benign nonmelanotic CNS tumour (patients 6, 9 and 13; see Table 2), all of which were removed by neurosurgery with no further sequelae. Others in this group had leptomeningeal disease, and while in four cases this was a progressive malignant process resulting in death, it is important to note that five patients have stable nonprogressive leptomeningeal disease (patients 3, 7, 8, 11 and 14; see Table 2), all involving focal lesions of unknown histology, which are, in some cases, quite extensive (Fig. 3). In all of these cases the lesions are currently asymptomatic. Furthermore, in patient 5 (aged 10 years at the time of writing) there is diffuse leptomeningeal disease indistinguishable radiologically from the four patients with progressive malignant process, which developed over the first year of life but which has not changed since then. Therefore, patients in group 2 have to be assessed on an individual basis, and our experience suggests that radiological progression or stability are useful guides to management. This is particularly useful in a condition where histology is often not reliable. New genetic tests on leptomeningeal lesions are likely to be helpful in the future to distinguish between benign and malignant lesions.

Comparison of our practice and results before and after the publication of guidelines on MRI in 2008 identified that we are doing significantly fewer scans as a proportion of new patient referrals since these guidelines were introduced (Fig. 2), whereas the detection rate of abnormalities in those having scans has not significantly changed (small increase) (Fig. 2). Importantly, the clinical phenotype profile of the cohort has not changed since the guidelines were changed. Therefore, we are confident that on the basis of this large prospective dataset the 2008 guidelines are fit for purpose, allowing detection of neurological abnormalities without performing unnecessary scans in very low‐risk individuals. However, as with all guidelines, there must always be room for clinical judgement.

## Discussion

We propose the continued use of the term ‘CMN syndrome’ for CMN with extracutaneous features, which we deem more appropriate than NCM due to nonmelanotic CNS lesions and a causative single gene defect for the cutaneous and neurological findings. This term also brings CMN into line with terminology used for other congenital naevi, where, for example, an epidermal naevus associated with extracutaneous features is termed ‘epidermal naevus syndrome’.

MRI of the CNS in children with CMN was originally started in 1988 at Great Ormond St Hospital as a research project in order to try to delineate the spectrum of disease in parallel with other centres, and to exploit the newly described sensitivity of MRI specific to melanin. This research project produced its first set of preliminary guidelines for the use of MRI in 2008, where the principal recommendation was to stop performing a routine scan for children with only a single CMN at birth; however, as is correct practice, these guidelines have now been audited to see if they continue to be fit for purpose. In the interim, we have continued to see new patients and have continued to expand our cohort of prospectively collected long‐term outcome data. This cohort is now large enough that we can start to answer the question of whether a single screening CNS MRI is a valuable clinical test in patients with CMN; in other words, does it alter our management?

The results presented herein show that routine MRI of the CNS in children with two or more CMN at birth, independent of projected adult size or site of the largest CMN, and undertaken within the first year of life (ideally within the first 6 months, as myelination obscures the signal for melanin), is the best predictor of neurodevelopmental abnormalities, seizures and the requirement for neurosurgery in childhood. It is, of course, not impossible that a child with a single CMN could have a neurological abnormality on MRI; however, comparison of our data from before and after the publication of the 2008 guidelines has found that while the percentage of children scanned has significantly decreased, there has been no significant change in the percentage of abnormal scans. This suggests that the guidelines are currently fit for purpose in terms of not missing large numbers of children with serious intracranial pathology.

Clinical management was altered substantially by the radiological results. Firstly, brain and spinal tumours were resected when causing compression, and follow‐up scans were used to monitor recurrence. Secondly, ventriculoperitoneal shunts were inserted where there was hydrocephalus, and follow‐up scans used to monitor position and progression/resolution. Thirdly, children with leptomeningeal disease were monitored by MRI, and where it progressed a biopsy was taken to look for melanoma histologically and genetically. Lastly, where intraparenchymal melanosis alone was detected the children were seen by a paediatric neurologist at least once, and by a developmental neurologist on an annual basis, and where intervention was required (e.g. speech therapy, occupational therapy, behavioural intervention), this was instituted promptly as for any child with these clinical problems.

For many years we have recognized that the clinical outcome of children with ‘symptomatic’ neurological disease has not necessarily been poor, despite the majority of publications on CMN still professing this idea. Therefore, the secondary aim of this study was an attempt at subclassification of radiological abnormalities, and to correlate this with outcome measures. This has identified a high‐risk group frequently requiring neurosurgery in early childhood, and possibly at higher risk of death from CNS melanoma (although this has not been modelled statistically as the numbers of deaths are small) (group 2), while providing reassurance that the most common finding of intraparenchymal melanosis alone is a nonmalignant condition, even when symptomatic (group 1).

**Figure 4:**
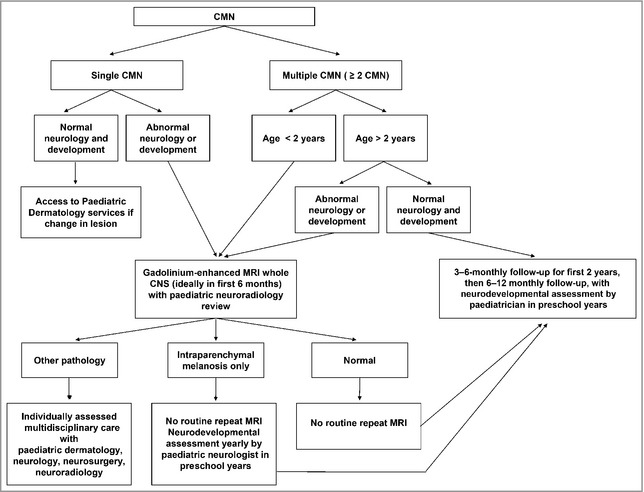
Great Ormond Street Hospital management guidelines for children with congenital melanocytic naevi (CMN). MRI, magnetic resonance imaging; CNS, central nervous system.

We propose that those with normal MRI results do not require either routine repeat MRI or formal neurodevelopmental follow‐up; that those in in group 1 do not require routine repeat MRI but do require neurodevelopmental follow‐up on an annual basis; and that those in group 2 should have regular repeat MRI and regular neurological follow‐up (Fig. 4). The periodicity of these interventions in group 2 would be decided by the multidisciplinary team, depending on the MRI findings; however, we would suggest that those with leptomeningeal disease need to monitored extremely closely initially as this disease can spread rapidly. However, if the findings are stable this intensity could be relaxed. Very importantly, any change in neurological status at any age should always trigger a repeat MRI, independent of the initial MRI findings.

## Supporting information


**Appendix S1.** Regression analysis.
