# Supplementary material for: Classification of neurological abnormalities in children with congenital melanocytic naevus syndrome identifies magnetic resonance imaging as the best predictor of clinical outcome
Source: Br J Dermatol. 2015 Aug 27;173(3):739–50. doi: 10.1111/bjd.13898 (PMC4737261; doi:10.1111/bjd.13898)
Supplement: Supplementary file 1 — Appendix S1. Regression analysis. [file BJD-173-739-s001.docx]

**Supplementary material, Waelchli et al, BJD 2015**

Regression analysis output from statistics programme SPSS version 21 for the three outcome measures as binary variables (seizures, neurodevelopmental abnormalities, and requirement for neurosurgery). In all three cases the logistic regression with only CMN projected adult size as the independent variable is shown first, then MRI result is added as a second independent variable, and then the MRI result is modeled alone where the projected adult size had been shown to be non-significant. The projected adult size is entered as a non-categorical variable for ease of interpretation, however the same analysis with this variable as a categorical variable (as is more correct) produces the same overall result. In all three cases the addition of the MRI result renders the latter more significant than the projected adult size, and indeed removes the significance of the projected adult size association in two cases. The P values are indicated by the column labeled Sig. (significance), the odds ratio by the Exp(B) column, and the 95% confidence intervals of the odds ratio by the 95% CI for EXP(B).

**Dependent variable - Seizures**

| **Variables in the Equation** | | | | | | | | | |
| --- | --- | --- | --- | --- | --- | --- | --- | --- | --- |
|  | | B | S.E. | Wald | df | Sig. | Exp(B) | 95% C.I.for EXP(B) | |
|  |  |  |  |  |  |  |  | Lower | Upper |
| Step 1^a^ | CMNProjectedAdultSize | .693 | .214 | 10.491 | 1 | .001 | 2.001 | 1.315 | 3.044 |
|  | Constant | -5.322 | .945 | 31.728 | 1 | .000 | .005 |  |  |
| a. Variable(s) entered on step 1: CMNProjectedAdultSize. | | | | | | | | | |

| **Variables in the Equation** | | | | | | | | | |
| --- | --- | --- | --- | --- | --- | --- | --- | --- | --- |
|  | | B | S.E. | Wald | df | Sig. | Exp(B) | 95% C.I.for EXP(B) | |
|  |  |  |  |  |  |  |  | Lower | Upper |
| Step 1^a^ | CMNProjectedAdultSize | .375 | .229 | 2.676 | 1 | .102 | 1.454 | .928 | 2.278 |
|  | MRIResultAbnormal | 1.978 | .587 | 11.360 | 1 | .001 | 7.228 | 2.288 | 22.830 |
|  | Constant | -4.606 | .961 | 22.976 | 1 | .000 | .010 |  |  |
| a. Variable(s) entered on step 1: CMNProjectedAdultSize, MRIResultAbnormal. | | | | | | | | | |

| **Variables in the Equation** | | | | | | | | | |
| --- | --- | --- | --- | --- | --- | --- | --- | --- | --- |
|  | | B | S.E. | Wald | df | Sig. | Exp(B) | 95% C.I.for EXP(B) | |
|  |  |  |  |  |  |  |  | Lower | Upper |
| Step 1^a^ | MRIResultAbnormal | 2.593 | .535 | 23.523 | 1 | .000 | 13.375 | 4.690 | 38.146 |
|  | Constant | -3.574 | .414 | 74.560 | 1 | .000 | .028 |  |  |
| a. Variable(s) entered on step 1: MRIResultAbnormal. | | | | | | | | | |

**Dependent variable - Neurodevelopmental problems**

| **Variables in the Equation** | | | | | | | | | |
| --- | --- | --- | --- | --- | --- | --- | --- | --- | --- |
|  | | B | S.E. | Wald | df | Sig. | Exp(B) | 95% C.I.for EXP(B) | |
|  |  |  |  |  |  |  |  | Lower | Upper |
| Step 1^a^ | CMNProjectedAdultSize | .449 | .124 | 13.050 | 1 | .000 | 1.567 | 1.228 | 2.000 |
|  | Constant | -3.316 | .491 | 45.569 | 1 | .000 | .036 |  |  |
| a. Variable(s) entered on step 1: CMNProjectedAdultSize. | | | | | | | | | |

| **Variables in the Equation** | | | | | | | | | |
| --- | --- | --- | --- | --- | --- | --- | --- | --- | --- |
|  | | B | S.E. | Wald | df | Sig. | Exp(B) | 95% C.I.for EXP(B) | |
|  |  |  |  |  |  |  |  | Lower | Upper |
| Step 1^a^ | CMNProjectedAdultSize | .356 | .150 | 5.606 | 1 | .018 | 1.427 | 1.063 | 1.915 |
|  | MRIResultAbnormal | 1.091 | .435 | 6.287 | 1 | .012 | 2.978 | 1.269 | 6.987 |
|  | Constant | -3.168 | .588 | 29.083 | 1 | .000 | .042 |  |  |
| a. Variable(s) entered on step 1: CMNProjectedAdultSize, MRIResultAbnormal. | | | | | | | | | |

**Dependent variable - Neurosurgery required**

| **Variables in the Equation** | | | | | | | | | |
| --- | --- | --- | --- | --- | --- | --- | --- | --- | --- |
|  | | B | S.E. | Wald | df | Sig. | Exp(B) | 95% C.I.for EXP(B) | |
|  |  |  |  |  |  |  |  | Lower | Upper |
| Step 1^a^ | CMNProjectedAdultSize | .385 | .215 | 3.202 | 1 | .074 | 1.470 | .964 | 2.243 |
|  | Constant | -4.504 | .872 | 26.684 | 1 | .000 | .011 |  |  |
| a. Variable(s) entered on step 1: CMNProjectedAdultSize. | | | | | | | | | |

| **Variables in the Equation** | | | | | | | | | |
| --- | --- | --- | --- | --- | --- | --- | --- | --- | --- |
|  | | B | S.E. | Wald | df | Sig. | Exp(B) | 95% C.I.for EXP(B) | |
|  |  |  |  |  |  |  |  | Lower | Upper |
| Step 1^a^ | CMNProjectedAdultSize | -.218 | .260 | .705 | 1 | .401 | .804 | .483 | 1.338 |
|  | MRIResultAbnormal | 4.407 | 1.131 | 15.183 | 1 | .000 | 82.009 | 8.937 | 752.539 |
|  | Constant | -4.536 | 1.215 | 13.930 | 1 | .000 | .011 |  |  |
| a. Variable(s) entered on step 1: CMNProjectedAdultSize, MRIResultAbnormal. | | | | | | | | | |

| **Variables in the Equation** | | | | | | | | | |
| --- | --- | --- | --- | --- | --- | --- | --- | --- | --- |
|  | | B | S.E. | Wald | df | Sig. | Exp(B) | 95% C.I.for EXP(B) | |
|  |  |  |  |  |  |  |  | Lower | Upper |
| Step 1^a^ | MRIResultAbnormal | 4.263 | 1.060 | 16.170 | 1 | .000 | 71.029 | 8.893 | 567.322 |
|  | Constant | -5.421 | 1.002 | 29.253 | 1 | .000 | .004 |  |  |
| a. Variable(s) entered on step 1: MRIResultAbnormal. | | | | | | | | | |
